# Oncologic and Surgical Outcomes After Short-Course Neoadjuvant CAPOX Plus Bevacizumab in High-Risk Colorectal Liver Metastases

**DOI:** 10.3390/cancers18030521

**Published:** 2026-02-05

**Authors:** Yawen Dong, Madita Tschoegl, Florian Lehner, Jonas Santol, Francesca Notte, Mariel Gramberger, Mohammed Salem, Edanur Cenan, Rebecca Thonhauser, Thomas Hoblaj, Rosemarie Valenta, Birgit Gruenberger, Thomas Gruenberger

**Affiliations:** 1 Department of Surgery, HPB Center, Clinic Favoriten, Health Network Vienna and Sigmund Freud Private University, 1100 Vienna, Austria; yawen-dong@hotmail.com (Y.D.); madita-magdalena.tschoegl@gesundheitsverbund.at (M.T.); telllehner@gmail.com (F.L.); jonas.santol@gesundheitsverbund.at (J.S.); francesca.notte@gesundheitsverbund.at (F.N.); mariel.gramberger@gesundheitsverbund.at (M.G.); mohamed.salem@gesundheitsverbund.at (M.S.); edanur.cenan@gesundheitsverbund.at (E.C.); rebecca.thonhauser@gesundheitsverbund.at (R.T.); thomas.hoblaj@gesundheitsverbund.at (T.H.); 2 Department of Radiology, Clinic Favoriten, Health Network Vienna, 1100 Vienna, Austria; rosmarie.valenta@gesundheitsverbund.at; 3 Department of Oncology, University Hospital Wiener Neustadt, 2700 Wiener Neustadt, Austria; birgit.gruenberger@wienerneustadt.lknoe.at

**Keywords:** response evaluation, morphologic pattern, tumor regression grade, Fong score, survival outcomes, washout period

## Abstract

Background: The optimal duration of neoadjuvant therapy for high-risk colorectal liver metastases (CRLM) remains debated. While prolonged chemotherapy may enhance response, it also increases toxicity and risks delaying potentially curative resection. These considerations have raised the question whether a short-course neoadjuvant strategy might achieve sufficient oncologic selection and response while minimizing treatment-related morbidity. Methods: Patients with synchronous or metachronous CRLM who received two cycles of neoadjuvant CAPOX plus bevacizumab followed by curative-intent liver resection treated between 2014 and 2024 at Health Network Vienna, Austria, were included. Clinicopathologic characteristics, treatment tolerability, response assessments (biochemical, radiologic, and pathologic), and survival outcomes were collected and analyzed. Results: A total of 57 patients were included (65% synchronous, 35% metachronous), with the rectum being the most frequent primary tumor site (45.6%). Most liver lesions were <5 cm (84.2%), and 47% had bilobar disease. Minor hepatectomy was performed in 65% of cases, predominantly via open surgery (72%). Grade ≥3 treatment-related adverse events occurred in 6 patients (10.6%), mainly neutropenia and diarrhea. Biochemically, 53.7% achieved >50% tumor marker reduction. Radiologic assessment showed partial response in 31.6% and complete response in 1.7%. Pathologic evaluation revealed TRG 3 as the most common finding (57.1%), followed by TRG 2 in 22.5%. Subgroup analyses demonstrated significantly improved OS and RFS in patients receiving adjuvant therapy and in those with tumors < 5 cm. Conclusion: A two-cycle, short-course regimen of CAPOX plus bevacizumab proved both effective and safe in high-risk CRLM, achieving meaningful biochemical, radiologic, and pathologic responses with acceptable toxicity. This abbreviated approach allowed delivery of neoadjuvant therapy while limiting cumulative treatment-related toxicity, supporting its feasibility as a neoadjuvant strategy in selected high-risk CRLM patients.

## Simple Summary

The optimal duration of chemotherapy before surgery for high-risk colorectal cancer liver metastases is a subject of ongoing debate. Prolonged treatment can lead to significant toxicity and delay potentially curative surgery. This study investigated whether a shorter, two-cycle chemotherapy regimen administered before surgery could achieve clinically relevant oncologic response with improved tolerability. Our findings from 57 patients indicate this abbreviated approach was safe and feasible, demonstrating meaningful biochemical, radiologic, and pathologic tumor responses with an acceptable toxicity profile. This suggests that a less intensive neoadjuvant strategy could be a viable option for carefully selected patients with high-risk colorectal liver metastases, potentially optimizing patient outcomes and facilitating timely surgical intervention. These insights may help guide future treatment decisions for this complex clinical scenario.

## 1. Introduction

The management of colorectal liver metastases (CRLM) remains a complex clinical challenge, even in the era of multimodal therapy. Despite technically curative liver resection, approximately 70% of patients relapse, underscoring the urgent need for strategies that improve systemic disease control and long-term outcomes. The integration of systemic therapy with surgical resection has therefore become standard in patients with resectable or potentially resectable disease: neoadjuvant (or perioperative) chemotherapy aims not only to downstage tumor burden and convert borderline cases to resectability, but also to target micrometastatic disease beyond the liver [1,2,3].

Selection of patients for neoadjuvant therapy is guided by the recognition that certain disease characteristics confer a markedly elevated risk of early recurrence. High-risk features include (i) more than four liver metastases, (ii) lesion diameter greater than 5 cm, (iii) synchronous presentation of liver metastases (i.e., diagnosed at the same time as the primary tumor) or metastatic disease emerging within one year after primary tumor resection, (iv) lymph node-positive primary tumors, (v) elevated carcinoembryonic antigen (CEA) levels pre-treatment, (vi) bilobar intra-hepatic spread or invasion of intrahepatic vascular structures, and (vii) resection scenarios where achieving an R0 margin is unlikely or where adequate residual functional liver volume is compromised. These features collectively identify patients at high risk of recurrence within 6 to 12 months of resection, and thus prime candidates for intensified neoadjuvant strategies [4,5,6].

Among the many chemotherapy combinations studied in this setting, the regimen of capecitabine plus oxaliplatin (CAPOX) in combination with the anti-VEGF monoclonal antibody bevacizumab has emerged as a compelling option for high-risk CRLM patients. Several key features support its adoption: first, high objective response rates (ORR), up to 78% in high-risk cohorts, and conversion to resectability in as many as 40% of initially unresectable cases; in more favorable (potentially resectable) populations ORRs of ~66% and R0 resection rates of ~97% have been documented [7,8]. In high-risk molecular subsets such as RAS-mutant CRLM, adding bevacizumab to oxaliplatin-based therapy markedly improved ORR, R0 resection rates, progression-free survival (PFS) and overall survival (OS) compared with chemotherapy alone [9,10,11]. Second, the combination offers a favorable perioperative safety profile: although oxaliplatin-based regimens carry a risk of chemotherapy-associated liver injury (CALI) such as sinusoidal obstruction syndrome (SOS), the addition of bevacizumab appears protective, reducing the incidence of SOS and postoperative liver failure [12,13,14]. When bevacizumab is suspended at least 5–6 weeks before hepatectomy, the regimen does not increase wound-healing complications or perioperative morbidity [8,15]. Third, long-term follow-up data suggest a survival advantage: in the ASSO-LM1 trial, patients completing both neoadjuvant and adjuvant CAPOX plus bevacizumab achieved a median OS of 59.1 months compared to 30.8 months for those who had neoadjuvant therapy alone [16].

Nonetheless, despite this solid evidence for neoadjuvant CAPOX and bevacizumab in the 4–6 cycle (or more) preoperative setting, the optimal duration of neoadjuvant therapy in high-risk resectable CRLM remains undefined. All published studies thus far have used 4 to 6 cycles prior to surgery. None have evaluated an *extra-short* regimen of only two cycles. In this context, we postulated that a brief window of two cycles of neoadjuvant CAPOX plus bevacizumab might offer distinct advantages: it could enable rapid assessment of tumor biology and early treatment response, limit cumulative CALI, minimize exposure to systemic therapy before surgery, and importantly avoid delaying definitive resection. We therefore examined the feasibility, surgical and oncological outcomes, including postoperative morbidity, OS, RFS, and treatment response, of this extra-short neoadjuvant strategy in high-risk CRLM patients.

## 2. Methods

### 2.1. Study Design and Patient Selection

Patients who received two cycles of neoadjuvant chemotherapy with capecitabine and oxaliplatin (CAPOX) in combination with bevacizumab prior to liver resection were retrospectively identified through institutional chart review. The study cohort included patients treated between April 2014 and November 2024 at Health Network Vienna. Both synchronous and metachronous CRLM were eligible for inclusion. Patients who underwent simultaneous colorectal and liver resection as well as staged procedures were included. High-risk CRLMs in this study were defined using a pragmatic, multidisciplinary, clinically driven approach that prioritized synchronous metastatic disease, rather than risk stratification based solely on formal prognostic scores such as the Fong score.

### 2.2. Chemotherapy Regimen

The decision to administer a short-course neoadjuvant regimen limited to two cycles was based on multidisciplinary evaluation, incorporating tumor biology, anticipated surgical complexity, patient-specific factors including comorbidities and treatment tolerance, and the intent to avoid unnecessary delays to surgical resection. All patients received a total of two cycles of neoadjuvant chemotherapy consisting of capecitabine and oxaliplatin (CAPOX) in combination with bevacizumab. Bevacizumab 7.5 mg/kg was administered by intravenous infusion, and oxaliplatin 130 mg/m^2^ was infused over a 2-h period; both agents were given on day 1 of each 3-week cycle. Capecitabine 1000 mg/m^2^ was administered orally twice daily on days 1 through 14 of each 3-week cycle. Surgery was planned no earlier than five weeks after the final bevacizumab dose to minimize the risk of wound-healing complications [8].

### 2.3. Assessment of Treatment Response and Toxicity

Treatment response was assessed across three categories: biochemical, radiologic, and pathologic. *Biochemical* response was defined as a >50% decline in serum CEA or CA19-9 levels relative to baseline [17,18]. *Radiologic* response was evaluated according to the Response Evaluation Criteria in Solid Tumors (RECIST 1.1), supplemented by an assessment of morphologic changes in CRLM [19]. Specifically, the development of a cystic transformation pattern following neoadjuvant therapy was evaluated according to CT-based morphologic pattern changes, classified into morphology groups 1–3 in accordance with the system proposed by Chun et al. [20,21]. Morphology group 1 was defined by homogeneous hypoattenuation with a sharp tumor–liver interface and complete resolution of any initial peripheral rim enhancement; morphology group 2 was characterized by mixed attenuation with a variable tumor–liver interface and partial resolution of peripheral rim enhancement if initially present; and morphology group 3 was defined by heterogeneous attenuation with an ill-defined tumor–liver interface and persistent or newly present peripheral rim enhancement. Morphologic response was defined as optimal when the metastasis changed from group 3 or 2 to group 1, incomplete when the group changed from 3 to 2, and absent when the group remained unchanged or increased. *Histopathologic* response after neoadjuvant therapy was evaluated using the Rubbia-Brandt Tumor Regression Grade (TRG) system with the assessment of the relative amount of residual viable tumor compared with chemotherapy-induced fibrosis [22,23,24]. Lesions were classified into five grades (TRG 1–5), ranging from complete tumor regression with predominantly fibrotic replacement (TRG 1) to an absence of histologic treatment effect, marked by extensive viable tumor and lack of fibrosis (TRG 5). The intermediate grades—TRG 2, TRG 3, and TRG 4—correspondingly denote major, partial, and minimal regression [25]. For patients with multiple metastases, the lowest TRG (best response) was recorded for analysis.

### 2.4. Surgical and Oncologic Outcomes

The choice of surgical approach (open vs. minimally invasive) was based on tumor burden, anatomical complexity, patient comorbidities, and anticipated extent of resection, in line with institutional practice. The extent of liver resection was classified according to the Brisbane 2000 nomenclature of the International Hepato-Pancreato-Biliary Association (IHPBA), with minor resections defined as the removal of fewer than three Couinaud segments and major resections as the resection of three or more segments [26]. Perioperative morbidity was classified according to the Clavien–Dindo grading system [27]. Operative variables, including the extent of hepatectomy, operative time, and postoperative length of hospital stay, were recorded. Oncologic outcomes included overall survival (OS) and recurrence-free survival (RFS). Subgroup analyses were also performed, among others using radiologic response categories and receipt of adjuvant therapy as stratification parameters for outcome evaluation. To further characterize factors associated with oncologic outcomes, prognostic assessment included calculation of the Fong Clinical Risk Score, which is based on five established preoperative factors: node-positive primary tumor, a disease-free interval of <12 months between primary tumor resection and diagnosis of liver metastases, the presence of more than one liver metastasis, a largest metastatic lesion measuring > 5 cm, and a preoperative CEA level > 200 ng/mL.

Treatment-related adverse events (TRAEs) were systematically documented and graded according to the Common Terminology Criteria for Adverse Events (CTCAE), version 5.0 [28]. Dose reductions, treatment delays, or therapy interruptions were performed at the discretion of the treating medical oncologist based on the severity of toxicity, overall clinical status, and individual patient tolerance.

### 2.5. Ethical Considerations

The study was carried out in compliance with institutional guidelines for clinical research at Clinic Favoriten, Health Network Vienna, and in accordance with the Declaration of Helsinki. Due to the retrospective design, the requirement for informed consent was waived.

### 2.6. Statistical Analysis

Continuous variables were summarized as medians with interquartile ranges (IQRs) and compared using the Mann–Whitney U or Kruskal–Wallis tests, as appropriate. Categorical variables were expressed as counts and percentages and compared using the χ^2^ test or Fisher’s exact test.

OS was defined as the time from the initial diagnosis of CRLM to death or the date of last follow-up. Recurrence-free survival (RFS) was defined from the date of curative-intent hepatic resection to radiologically or histologically confirmed recurrence or death, whichever occurred first. Patients without an event were censored at their last follow-up.

Survival curves were estimated using the Kaplan–Meier method, and comparisons between groups were performed with the log-rank test. Predictors of categorical outcomes were analyzed using univariable and multivariable logistic regression models. Cox proportional hazards regression models were used to evaluate the association between clinicopathologic variables and overall and recurrence-free survival, with covariates selected a priori based on clinical relevance. All statistical analyses were performed using IBM SPSS Statistics, version 28.0 (IBM Corp., Armonk, NY, USA). All reported *p* values are two-sided, and results were considered statistically significant at *p* < 0.05.

## 3. Results

### 3.1. Baseline Characteristics of the Study Population

**Figure 1:**
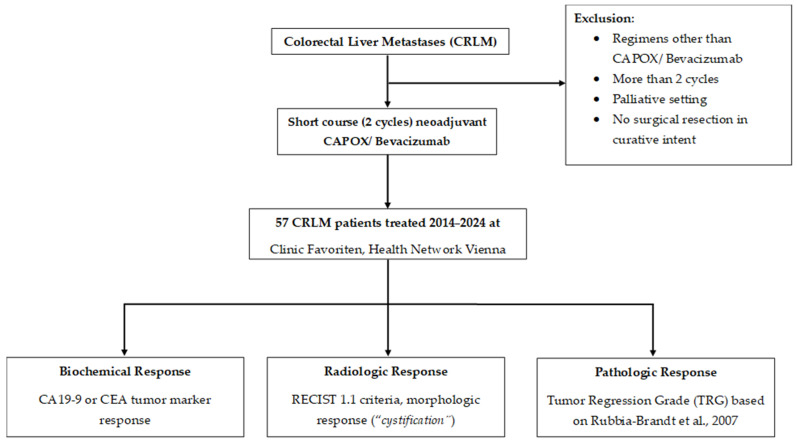
Flowchart of the patient cohort, including applied exclusion criteria and the three primary outcomes: biochemical, radiologic, and pathologic response. CA19-9 Carbohydrate Antigen 19-9, CEA Carcinoembryonic Antigen, CAPOX Capecitabine plus Oxaliplatin [22].

The flowchart of the study cohort detailing patient inclusion and exclusion criteria as well as the primary outcomes of interest is depicted in Figure 1. Of the entire patient cohort with CRLM (n = 57), the majority were male (66.7%), with a median age of 66.5 years and a median body mass index (BMI) of 26.0 kg/m^2^ (Table 1). Patients were predominantly classified as ASA II (43.9%) or ASA III (50.8%). In terms of tumor markers, the median pre-treatment CA19-9 level was 24.6 U/mL, which decreased to 20.0 U/mL pre-operatively. Notably, median pre-treatment CEA decreased markedly from 16.9 ng/mL to 4.5 ng/mL prior to surgery. Preoperative liver function assessment using the APRI+ALBI score yielded a median value of −2.5 in the current cohort. Clinical characteristics of CRLM revealed that synchronous disease was more common (64.9%) than metachronous (35.1%). The primary tumor was most frequently located in the rectum (45.6%), followed by left-sided (28.1%) and right-sided (26.3%) colon. At diagnosis, most CRLM lesions were ≤5 cm (84.2%), with the majority of patients presenting with 2–4 lesions (56.1%). Nearly half of the cohort (49.1%) had bilobar disease. Overall, these findings indicate that the cohort was enriched for high-risk disease features, with frequent bilobar involvement, a clinically relevant lesion burden, and a predominance of synchronous metastatic presentation.

### 3.2. Clinicopathological Parameters

The majority underwent minor liver resection (64.9%), with major resections accounting for 35.1% (Table 2). All patients adhered to the prespecified minimum ≥5-week washout period prior to surgery, with no deviations observed. The predominant surgical approach was open (71.9%), while 22.8% were performed minimally invasively, and 5.3% required conversion. Simultaneous resection of the primary tumor and CRLM was undertaken in 22.8% of patients. Most patients (82.5%) underwent surgery for primary CRLM, with 17.5% undergoing surgery for recurrent CRLM. Intraoperatively, a Pringle maneuver was performed in 36.8% of cases, with a median Pringle time of 25 min. The median operative time was 184 min. Postoperative recovery was generally favorable, with a median ICU stay of 1 day and a median total length of hospital stay of 7 days. Postoperative morbidity, assessed by Clavien-Dindo classification, showed that 64.9% of patients experienced no complications (Grade 0), while 12.2% had Grade II complications, 10.5% had Grade III, 1.8% had Grade IV, and 5.3% had Grade V. Molecular analysis revealed RAS mutations in 35.1% of patients and BRAF mutations in 5.3%. All included patients exhibited proficient mismatch repair (pMMR), corresponding to microsatellite-stable (MSS) disease. Pathologic examination of the resected specimens showed that the most common primary tumor T stage was T3 (52.6%). Lymph node status was categorized as N0 in 28.1%, N1 in 29.8%, and N2 in 19.3%. Tumor grading predominantly showed G2 (64.9%) and G3 (22.8%) tumors. The median decrease in tumor size following neoadjuvant therapy was 23.9%. Adjuvant therapy was administered to 63.2% of patients. Among those, 61.1% received CAPOX or FOLFOX in combination with Bevacizumab, while 33.3% received CAPOX or FOLFOX alone. Recurrence was observed in 64.9% of the cohort, with the majority of recurrences being hepatic (67.6%), followed by pulmonary (24.3%) and locoregional at the primary tumor site (8.1%).

### 3.3. Biochemical, Radiologic and Pathologic Response

**Figure 2:**
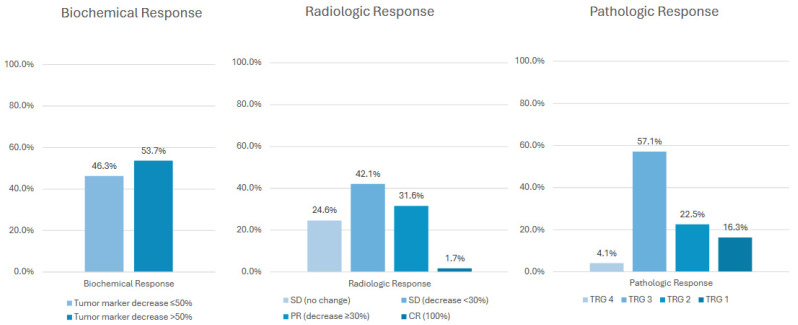
Overview of treatment response evaluated across three modalities: biochemical response (defined as ≥50% reduction in CEA or CA19-9 levels), radiologic response according to RECIST version 1.1, and pathologic response determined by tumor regression grade (TRG).

In terms of biochemical response, 53.7% of patients had a >50% decrease in tumor markers. The remaining 46.3% showed a ≤50% reduction or no change from baseline, and importantly, none experienced an increase following therapy. Radiologic response evaluation demonstrated stable disease (no change) in 24.6% of patients. A minor response (stable disease with <30% decrease) was noted in 42.1%, and a partial response (≥30% decrease) in 31.6%. One patient (1.7%) achieved a complete response. With regard to pathologic response, TRG 3 was the most frequent finding, observed in 57.1% of cases (Figure 2). In terms of morphologic response upon imaging, in the pre-therapeutic setting 48 patients (84.2%) were classified as morphology group 3, whereas 3 patients (5.3%) were assigned to morphology group 2 and 1 patient (1.8%) to morphology group 1; morphologic classification was not unavailable in five patients. Following neoadjuvant treatment, 28 of 52 evaluable patients (53.8%) achieved an optimal morphologic response, whereas 24 patients (46.2%) demonstrated an incomplete or absent response (Supplementary Figure S1).

### 3.4. Treatment-Related Adverse Events and Oncologic Outcomes

Overall, the short duration of neoadjuvant therapy was associated with a favorable safety profile. Severe adverse events (SAEs) were reported in 6 patients (10.5%), consisting of four cases of grade ≥3 diarrhea and two cases of grade ≥3 neutropenia.

**Figure 3:**
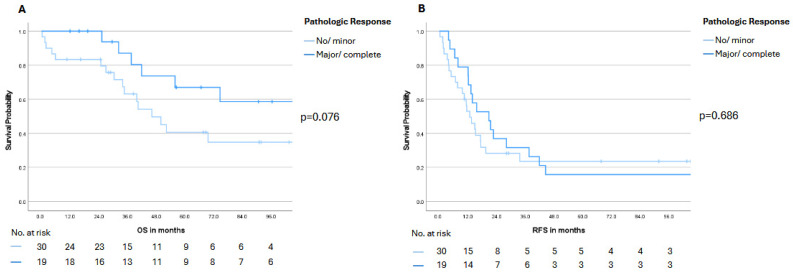
Overall survival (OS, panel (**A**)) and recurrence-free survival (RFS, panel (**B**)) stratified by pathologic response according to the tumor regression grade (TRG) system by Rubbia-Brandt et al. [22]. No/minor response was defined as TRG 5, 4 or 3, whereas major/complete response was defined as TRG 2 or 1. Numbers at risk are shown below the Kaplan–Meier curves. Statistical significance was defined as a *p*-value < 0.05.

Kaplan–Meier survival curves stratified by pathologic response demonstrated numerically longer OS in patients achieving a major or complete response (TRG 1–2) compared with those exhibiting no or minor pathologic response. Median OS was not reached in the major/complete response group, whereas it was 46.1 months (95% CI 32.4–59.8) in the no/minor response group; however, this difference did not reach statistical significance (*p* = 0.076; Figure 3A). Similarly, RFS analysis revealed a numerically longer median RFS for the major/complete response group (20.7 months, 95% CI 9.9–31.5) compared to the no/minor response group (12.7 months, 95% CI 8.1–17.3), although this difference did not achieve statistical significance (*p* = 0.686; Figure 3B). Furthermore, stratification by individual TRG categories showed no significant differences between grades with respect to either OS or RFS (Supplementary Figure S2).

**Figure 4:**
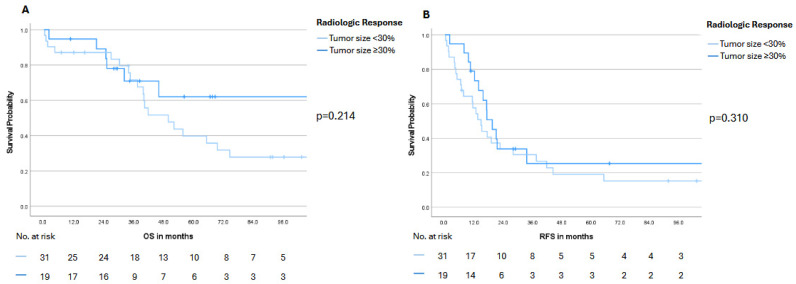
Overall survival (OS, panel (**A**)) and recurrence-free survival (RFS, panel (**B**)) stratified by radiologic response according to RECIST 1.1, using a ≥30% reduction in tumor size as the cutoff. Numbers at risk are displayed below the Kaplan–Meier curves. Statistical significance was defined as a *p*-value < 0.05.

When stratified by radiologic response according to RECIST 1.1, both OS and RFS were comparable between patients with partial response (tumor size reduction ≥ 30%) and those with stable disease (tumor size reduction < 30%) (Figure 4). Furthermore, radiologic morphologic response did not translate into a significant survival benefit in this patient subset (Supplementary Figure S3). Similarly, stratification by the presence of bilobar disease did not reveal a statistically significant difference in OS or RFS, although patients without bilobar involvement demonstrated a numerically more favorable outcome (Supplementary Figure S4). Additionally, exploratory subgroup analyses by primary tumor location and KRAS/BRAF mutation status did not reveal statistically significant differences and are reported in Supplementary Figures S5 and S6.

**Figure 5:**
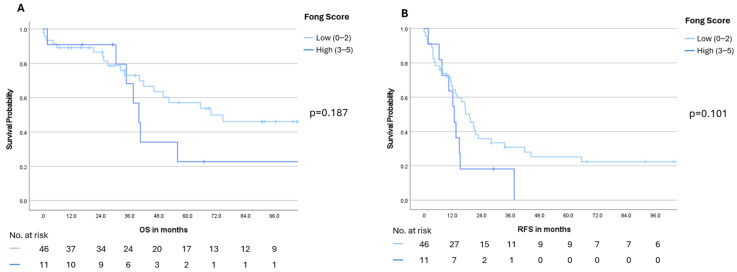
Overall survival (OS, panel (**A**)) and recurrence-free survival (RFS, panel (**B**)) stratified by Fong Risk Score low (0–2) vs. high (3–5). Numbers at risk are displayed below the Kaplan–Meier curves. A *p*-value < 0.05 was considered statistically significant.

**Figure 6:**
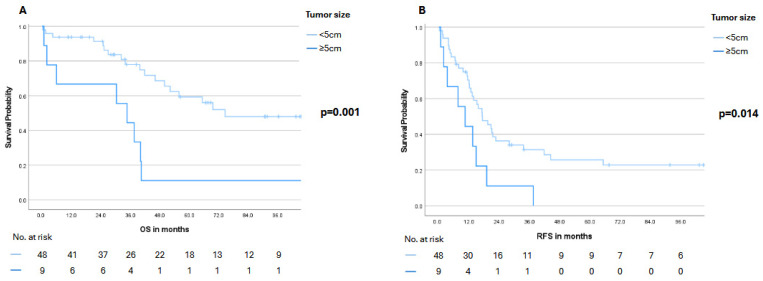
Overall survival (OS, panel (**A**)) and recurrence-free survival (RFS, panel (**B**)) stratified by tumor size (<5 cm vs. ≥5 cm). Numbers at risk are displayed below the Kaplan–Meier curves. A *p*-value < 0.05 was considered statistically significant.

**Figure 7:**
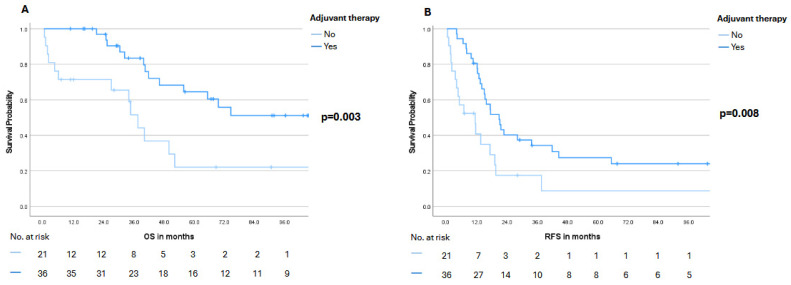
Overall survival (OS, panel (**A**)) and recurrence-free survival (RFS, panel (**B**)) stratified by receipt of adjuvant therapy (yes vs. no). Numbers at risk are displayed below the Kaplan–Meier curves. A *p*-value < 0.05 was considered statistically significant.

Notably, stratification by the Fong Clinical Risk Score (low: 0–2 vs. high: 3–5) revealed a pronounced numerical advantage for the low-risk group in both OS and RFS. Median OS was 69.6 months (95% CI: not reached) in the low-risk group compared with 39.8 months (95% CI: 35.6–44.0) in the high-risk group (*p* = 0.187). A similar pattern was observed for RFS, with a median of 19.0 months (95% CI: 13.7–24.3) versus 12.7 months (95% CI: 9.5–15.9), respectively (*p* = 0.101) (Figure 5). Moreover, patients with the largest metastatic lesion measuring <5 cm experienced significantly better survival outcomes. Median OS was 74.6 months (95% CI: not reached) in this subgroup, compared with 34.6 months (95% CI: 22.0–47.2) in patients with lesions ≥5 cm (*p* = 0.001). A similar trend was noted for RFS, with a median of 17.2 months (95% CI: 11.2–23.2) versus 10.4 months (95% CI: 2.2–18.6), respectively (*p* = 0.014) (Figure 6). Finally, receipt of adjuvant therapy was also associated with a significant survival advantage: Median OS was not reached (95% CI: not reached) in the adjuvant group compared with 37.5 months (95% CI: 31.5–43.5) in patients without adjuvant therapy (*p* = 0.003). Median RFS likewise favored the adjuvant group, with 20.7 months (95% CI: 14.0–27.3) versus 11.2 months (95% CI: 2.6–19.8) (*p* = 0.008) (Figure 7). Notably, in the multivariable Cox proportional hazards model adjusting for clinically relevant covariates, receipt of adjuvant therapy emerged as the strongest protective factor associated with longer OS (HR 0.023, 95%CI 0.002–0.218; *p* = 0.001) and RFS (HR 0.093, 95%CI 0.025–0.352; *p* < 0.001) (Table 3).

## 4. Discussion

In this study, we examined the feasibility of a short-course perioperative regimen of CAPOX plus bevacizumab in patients with CRLM. Notably, the present cohort represents a high-risk population, based on established clinical criteria, with nearly half of patients presenting with bilobar disease, substantial lesion burden, and a predominance of synchronous metastatic disease. Despite its brief duration, two cycles of neoadjuvant CAPOX and bevacizumab were associated with consistent biochemical, radiologic, and pathologic response signals, while perioperative morbidity and mortality remained acceptable, with no apparent increase in bevacizumab-related surgical complications, contrary to some initial concerns [29]. The abbreviated treatment duration was associated with a favorable safety profile and a low incidence of adverse events in our cohort, supporting the feasibility of integrating bevacizumab within a concise, time-efficient neoadjuvant strategy. Ultimately, these findings suggest that this short-course strategy may be particularly advantageous in the preoperative setting, where achieving meaningful tumor response with limited treatment-related toxicity is essential. By demonstrating antitumor activity without the cumulative risks associated with prolonged systemic therapy, such as higher adverse event rates, reduced treatment tolerance, and potential delays to surgery, this abbreviated regimen may offer a favorable balance between efficacy and safety.

Recent reports have highlighted that perioperative CAPOX plus bevacizumab represents an effective and well-tolerated strategy for CRLM, harnessing the early cytoreductive effect of oxaliplatin-based therapy while limiting cumulative hepatotoxicity and perioperative risk. Oxaliplatin-containing regimens achieve meaningful responses within the first 8–12 weeks, after which further chemotherapy rarely adds tumor shrinkage but increases the likelihood of sinusoidal obstruction and steatohepatitis. The EORTC 40983 trial established perioperative FOLFOX as the reference regimen for resectable disease, improving progression-free survival (PFS) but showing no clear overall survival (OS) advantage from longer therapy, supporting a concise, response-adapted approach before surgery [30].

Another practical advantage of limiting neoadjuvant therapy to two cycles is the reduced risk of radiologic “disappearing” liver metastases, which, despite being undetectable on imaging, frequently persist as viable tumor deposits at surgery. Longer preoperative chemotherapy courses increase the likelihood of complete radiologic regression, complicating intraoperative localization and risking incomplete resection of residual disease. An abbreviated, response-guided approach allows tumor downsizing sufficient to assess chemosensitivity and facilitate safe resection, while maintaining reliable intraoperative detectability of all lesions. This balance between achieving cytoreduction and avoiding overtreatment further supports the use of an extra-short neoadjuvant CAPOX plus bevacizumab regimen in carefully selected patients [31,32].

From a tolerability standpoint, CAPOX plus bevacizumab demonstrates a favorable and predictable safety profile consistent with large phase III data. In the NO16966 trial, adding bevacizumab to oxaliplatin-based chemotherapy did not significantly worsen toxicity [33]. While CAPOX offers advantages, it is important to acknowledge that capecitabine, compared to intravenous 5-FU, can present distinct patient management challenges, particularly outside routine oncological monitoring, due to a higher incidence of hand-foot syndrome and potentially more pronounced diarrhea. For instance, CAPOX plus bevacizumab was associated with substantially lower rates of grade 3–4 neutropenia (12%) compared with FOLFOX plus bevacizumab (55–58%), but common grade 3–4 toxicities for CAPOX included fatigue and diarrhea. In the phase II BOXER study, lethargy (24%) and diarrhea (17%) were the most frequent, and subsequent dose adjustments improved tolerability [7]. Bevacizumab-related adverse events, such as hypertension, thromboembolism, and rare gastrointestinal perforation, remained infrequent and manageable with standard supportive care. These findings affirm the regimen’s suitability for the perioperative setting, particularly in shorter treatment durations, while highlighting areas for careful patient monitoring.

A defining strength of this approach lies in its structured administration incorporating a planned bevacizumab washout, which not only mitigates bleeding and wound-healing risks but has also been shown to contribute to a perioperative safety profile that is often superior to what might be expected. Prospective and phase II studies have consistently established that a 5–8-week interval between the final bevacizumab dose and liver resection ensures perioperative safety, with several papers demonstrating that perioperative morbidity and mortality tend to be lower rather than higher with bevacizumab. For instance, in the ASSO-LM1 trial, bevacizumab discontinuation 5 weeks preoperatively resulted in no increase in wound-healing or bleeding complications [16]. Likewise, the BOXER study reported no grade 3–4 perioperative events, and in the BECOME randomized trial (mFOLFOX6 plus bevacizumab), surgery at 6 weeks post-bevacizumab was not associated with grade ≥3 bleeding, anastomotic leakage, or wound infections [7,9]. In a single-center phase II study, only 6% of patients required perioperative blood transfusions, well below predefined safety thresholds, further confirming that short-course regimens incorporating bevacizumab are compatible with safe surgical outcomes and may even reduce overall perioperative risk [8].

Perioperative results from these studies are consistently encouraging. Reported postoperative morbidity rates remain low (14–22%), with no postoperative mortality in several trials. Moreover, a critical advantage of incorporating bevacizumab is its impressive protective effect against oxaliplatin-related sinusoidal obstruction syndrome (SOS), a significant driver of chemotherapy-associated liver injury (CALI) and post-hepatectomy liver failure (PHLF). In the ASSO-LM1 trial, only two cases of SOS were detected (5.7%), strongly supporting this protective role and suggesting a benefit beyond what CAPOX alone might offer [16].

Furthermore, prospective phase II and real-world datasets using CAPOX plus bevacizumab demonstrate high objective response rates of 70–80% in selected high-risk or borderline-resectable cohorts and conversion-to-resection rates of up to 40%. In nearly all studies, bevacizumab was held for ~5 weeks before surgery, reinforcing the short-course concept emphasizing early reassessment and timely resection. This strategy facilitates early radiologic evaluation (typically after 2–3 cycles) and proceeds directly to surgery once resectability is achieved, thus preventing overtreatment, limiting cumulative hepatotoxicity, and avoiding the pitfalls of delayed surgery [9,23,34]. Importantly, while CAPOX alone is an effective regimen, our findings and existing literature suggest that the addition of bevacizumab significantly enhances conversion and resection rates in patients with initially unresectable, liver-limited disease. This is evidenced by higher R0 resection and objective response rates in bevacizumab-containing regimens (e.g., mFOLFOX6 plus bevacizumab) compared with chemotherapy alone, despite relatively modest preoperative cycle numbers, underscoring that CAPOX alone may not achieve comparable oncologic outcomes [9].

Modern definitions of resectability in CRLM further support this paradigm. Resectability is now defined by the ability to achieve R0 or controlled R1vascular clearance while preserving an adequate future liver remnant (FLR) with intact inflow, outflow, and biliary drainage, rather than by lesion count or size. Contemporary guidance recommends FLR thresholds of ~20–25% in normal liver, ~30% after extensive chemotherapy or CALI, and ~40% in compromised parenchyma. When FLR is borderline, hypertrophy-inducing approaches such as portal vein embolization (PVE), portal vein ligation (PVL), or staged procedures (e.g., ALPPS) are applied, while parenchymal-sparing strategies remain preferred to preserve future treatment options. Regular multidisciplinary reassessment every 6–8 weeks ensures surgical timing coincides with peak tumor response [35,36].

This retrospective study has several limitations that need to be acknowledged. First, the limited sample size restricts statistical power, particularly for subgroup analyses, thereby limiting generalizability and necessitating cautious interpretation of these findings. Accordingly, subgroup results should be regarded as hypothesis-generating and interpreted as trends rather than definitive evidence, underscoring the need for prospective validation. Second, the absence of a contemporaneous control group precludes direct comparative effectiveness analyses. While our data support the feasibility of administering two cycles of neoadjuvant therapy in this selected population, they do not allow conclusions regarding its superiority relative to alternative treatment strategies. Third, as with all retrospective studies, residual confounding, missing data, and unmeasured variables cannot be fully excluded despite multivariable adjustment. Fourth, the definition of high-risk disease in this cohort warrants consideration. Although the majority of patients exhibited low Fong scores, this analysis intentionally prioritized individuals with synchronous metastatic colorectal cancer, introducing potential selection bias and limiting extrapolation to broader populations.

Taken together, these data support short-course perioperative CAPOX plus bevacizumab as a safe and feasible component of modern multimodal therapy for CRLM. The regimen was associated with (i) rapid and clinically relevant responses facilitating timely surgical resection; (ii) acceptable toxicity and perioperative safety profile; and (iii) implementation of an established bevacizumab washout interval, without an apparent increase in wound-healing or bleeding complications and with potential mitigation of oxaliplatin-associated hepatic injury. Collectively, these features suggest that a two-cycle short-course CAPOX plus bevacizumab regimen may represent a feasible and balanced perioperative approach within contemporary curative-intent strategies for CRLM, warranting further prospective evaluation.

## 5. Conclusions

This study evaluates the feasibility of a short-course perioperative regimen of CAPOX plus bevacizumab limited to two preoperative cycles in patients with CRLM. Despite its brief duration, this neoadjuvant approach was associated with consistent biochemical, radiologic, and pathologic response signals while maintaining acceptable treatment-related toxicity. By limiting cumulative exposure to systemic therapy, this strategy may help mitigate adverse events and avoid delays to surgery associated with prolonged treatment courses. Notably, perioperative morbidity and mortality did not appear to be increased with the incorporation of bevacizumab, including in high-risk CRLM patients. Overall, these findings support the feasibility of integrating bevacizumab within a concise, time-efficient neoadjuvant context. Further prospective studies are warranted to define the optimal patient selection and comparative benefit of this approach.

## Figures and Tables

**Table 1:** Baseline characteristics of the patient cohort (n = 57). NAT neoadjuvant therapy, BMI body mass index, ASA American Society of Anesthesiologists, CRLM colorectal liver metastasis, preOP pre-operative, APRI Aspartate Platelet Ratio Index, ALBI Albumin–Bilirubin Index. Data are shown as median (interquartile range) for continuous variables and as number (percentage) for categorical variables.

Variables	CRLM Patient Cohort (n = 57)
Sex:	
- Male	38 (66.7%)
- Female	19 (33.3%)
Age (years)	66.5 (57.6–74.0)
BMI (kg/m^2^)	26.0 (24.1–28.6)
ASA:	
- I	0 (0.0%)
- II	25 (43.9%)
- III	29 (50.8%)
- IV	3 (5.3%)
Pretreatment CA19-9 (U/mL)	24.6 (14.0–97.6)
PreOP CA19-9 (U/mL)	20.0 (11.7–35.3)
Pretreatment CEA (ng/mL)	16.9 (7.9–49.1)
PreOP CEA (ng/mL)	4.5 (2.2–11.0)
PreOP APRI+ALBI Score	−2.5 (−2.8–−2.2)
Timing of CRLM:	
- Synchronous	37 (64.9%)
- Metachronous	20 (35.1%)
Primary tumor:	
- Right-sided	15 (26.3%)
- Left-sided	16 (28.1%)
- Rectum	26 (45.6%)
Size of CRLM at diagnosis:	
- ≤5 cm	48 (84.2%)
- >5 cm	9 (15.8%)
Number of CRLM at diagnosis:	
- Solitary	12 (21.1%)
- 2–4	32 (56.1%)
- ≥5	13 (22.8%)
Bilobar disease	28 (49.1%)
Fong score:	
- Low (0–2)	46 (80.7%)
- High (3–5)	11 (19.3%)
Any severe adverse events (SAEs):	6 (10.5%)
- Diarrhea (grade ≥3)	4 (7.0%)
- Neutropenia (grade ≥3)	2 (3.5%)

**Table 2:** Perioperative outcomes and clinicopathological parameters of the patient cohort. MIS minimally invasive surgery, ICU intensive care unit, AJCC American Joint Committee on Cancer, NA not available, MMR mismatch repair, dMMR deficient mismatch repair, pMMR proficient mismatch repair, MSI-H microsatellite instability–high, MSS microsatellite stable.

Variables	CRLM Patient Cohort (n = 57)
Extent of liver resection:	
- Minor	37 (64.9%)
- Major	20 (35.1%)
Surgical approach:	
- Open	41 (71.9%)
- MIS	13 (22.8%)
- Conversion	3 (5.3%)
Simultaneous primary and CRLM resection	13 (22.8%)
Timing of liver resection:	
- Primary CRLM surgery	47 (82.5%)
- Recurrent CRLM surgery	10 (17.5%)
Pringle maneuver performed	21 (36.8%)
Pringle time (min)	25 (14–30)
OP time (min)	184 (155–225)
ICU stay (days)	1 (0–2)
Total length of stay (days)	7 (6–14)
Postoperative morbidity:	
- Clavien–Dindo 0	37 (64.9%)
- Clavien–Dindo I	3 (5.3%)
- Clavien–Dindo II	7 (12.2%)
- Clavien–Dindo III	6 (10.5%)
- Clavien–Dindo IV	1 (1.8%)
- Clavien–Dindo V	3 (5.3%)
RAS mutation	20 (35.1%)
BRAF mutation	3 (5.3%)
MMR status:	
- pMMR/MSS	57 (100%)
- dMMR/MSI-H	0 (0%)
Pathologic tumor (T) stage:	
- T0	2 (3.5%)
- T1	10 (17.5%)
- T2	5 (8.8%)
- T3	30 (52.6%)
- T4	5 (8.8%)
- NA	5 (8.8%)
Pathologic lymph node (N) status:	
- N0	16 (28.1%)
- N1	17 (29.8%)
- N2	11 (19.3%)
- NA	13 (22.8%)
Resection margin:	
- R0	54 (94.7%)
- R1	3 (5.3%)
Grading:	
- G1	2 (3.5%)
- G2	37 (64.9%)
- G3	13 (22.8%)
- NA	5 (8.8%)
Median decrease in tumor size (%)	23.9 (13.6–43.2)
Adjuvant therapy receipt	36 (63.2%)
Adjuvant therapy regimens:	
- CAPOX or FOLFOX	12 (33.3%)
- CAPOX or FOLFOX/Bevacizumab	22 (61.1%)
- Oxaliplatin/Bevacizumab	1 (2.8%)
- NA	1 (2.8%)
Recurrence	37 (64.9%)
Location of recurrence:	
- Hepatic	25 (67.6%)
- Pulmonary	9 (24.3%)
- Locoregional recurrence at primary tumor site	3 (8.1%)

**Table 3:** Multivariable Cox proportional hazards regression analysis for overall survival (OS) and recurrence-free survival (RFS) adjusting for clinically relevant covariates.

	Multivariable Cox Regression (OS)		Multivariable Cox Regression (RFS)	
Variables	HR (95% CI)	*p* Value	HR (95% CI)	*p* Value
Age	1.000 (0.909–1.101)	0.997	1.035 (0.979–1.093)	0.225
Sex (male)	8.200 (0.710–94.702)	0.092	4.379 (1.253–15.306)	0.021
Primary tumor location				
- Right-sided colon	Reference		Reference	
- Left-sided colon	2.883 (0.214–38.887)	0.425	0.657 (0.136–3.176)	0.602
- Rectum	5.771 (1.002–33.252)	0.050	2.696 (0.806–9.019)	0.107
Number of metastases				
- 1	Reference		Reference	
- 2–5	44.459 (1.209–1635.487)	0.039	2.374 (0.366–15.394)	0.365
- >5	17.954 (0.249–1292.666)	0.186	6.179 (0.502–72.074)	0.155
Tumor size	0.134 (0.005–3.945)	0.244	0.856 (0.095–7.724)	0.890
Fong score	4.163 (0.199–87.300)	0.358	3.894 (0.773–19.615)	0.099
Bilobar disease	0.665 (0.061–7.299)	0.739	0.592 (0.121–2.888)	0.517
KRAS/BRAF mutation	20.805 (2.531–171.048)	0.005	2.894 (0.942–8.891)	0.063
Tumor regression grade (TRG)	0.186 (0.023–1.521)	0.117	1.099 (0.307–3.937)	0.885
Biochemical response	0.676 (0.098–4.656)	0.691	0.151 (0.029–0.784)	0.025
Radiologic response	2.749 (0.551–13.720)	0.218	1.255 (0.415–3.791)	0.688
Morphologic response	7.124 (0.790–64.246)	0.080	0.445 (0.106–1.872)	0.269
Adjuvant therapy receipt	0.023 (0.002–0.216)	0.001	0.093 (0.025–0.352)	<0.001

## Data Availability

The data presented in this study are available from the corresponding author upon reasonable request due to institutional data protection policies.

## Supplementary Materials

The following supporting information can be downloaded at: https://www.mdpi.com/article/10.3390/cancers18030521/s1, Figure S1: Sankey diagram illustrating transitions in morphologic pattern from pre-treatment to post-treatment according to the CT-based Chun classification (groups 1–3). Figure S2: Overall survival (OS, panel A) and recurrence-free survival (RFS, panel B) stratified by pathologic response according to the tumor regression grade (TRG) system by Rubbia-Brandt et al. Figure S3: Overall survival (OS, panel A) and recurrence-free survival (RFS, panel B) stratified by morphologic response on computed tomography according to the Chun classification. Figure S4: Overall survival (OS, panel A) and recurrence-free survival (RFS, panel B) stratified by presence of bilobar disease. Figure S5: Overall survival (OS, panel A) and recurrence-free survival (RFS, panel B) stratified by the location of primary tumor (right-sided colon, left-sided colon, rectum). Figure S6: Overall survival (OS, panel A) and recurrence-free survival (RFS, panel B) stratified by the KRAS or BRAF mutation.
