# Supplementary material for: Oncologic and Surgical Outcomes After Short-Course Neoadjuvant CAPOX Plus Bevacizumab in High-Risk Colorectal Liver Metastases
Source: Cancers (Basel). 2026 Feb 5;18(3):521. doi: 10.3390/cancers18030521 (PMC12896431; doi:10.3390/cancers18030521)
Supplement: Supplementary file 1 [file cancers-18-00521-s001.zip › cancers-4083004-supplementary.pdf]

Supplementary Materials:

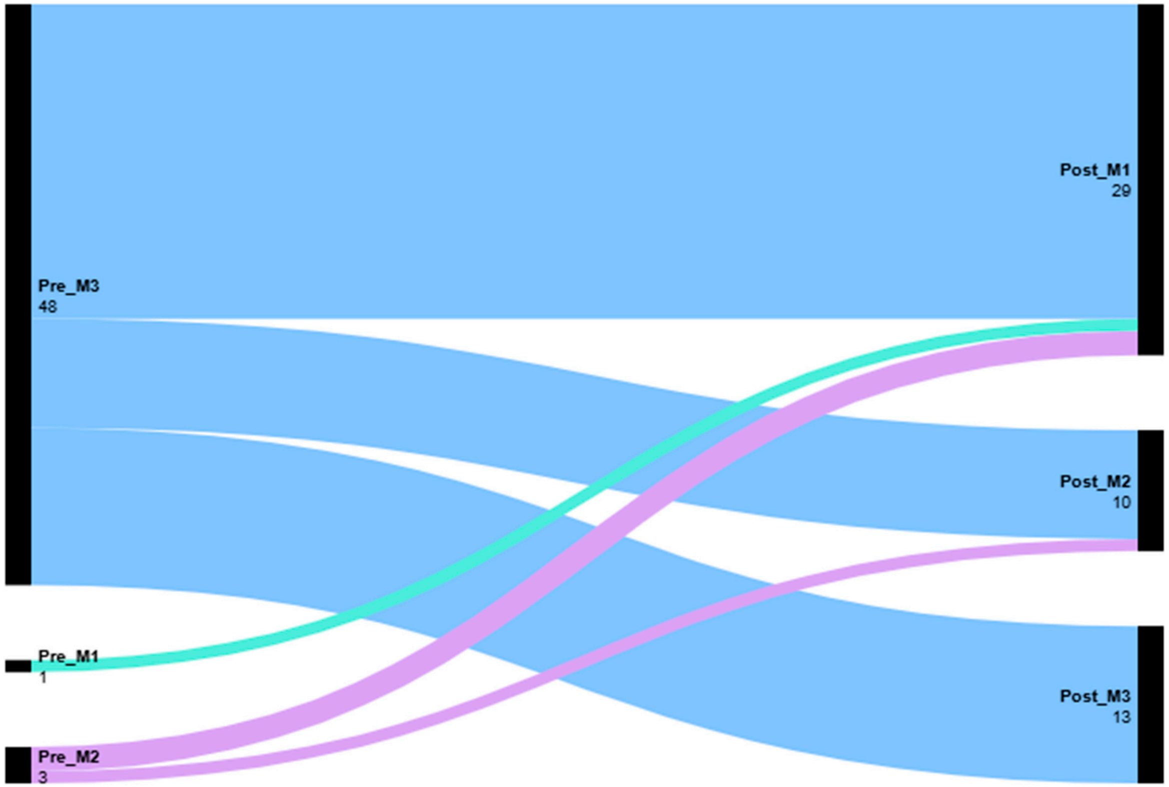

Supplementary Figure S1. Sankey diagram illustrating transitions in morphologic pattern from pre-treatment to post-treatment according to the CT-based Chun classification (groups 1–3).

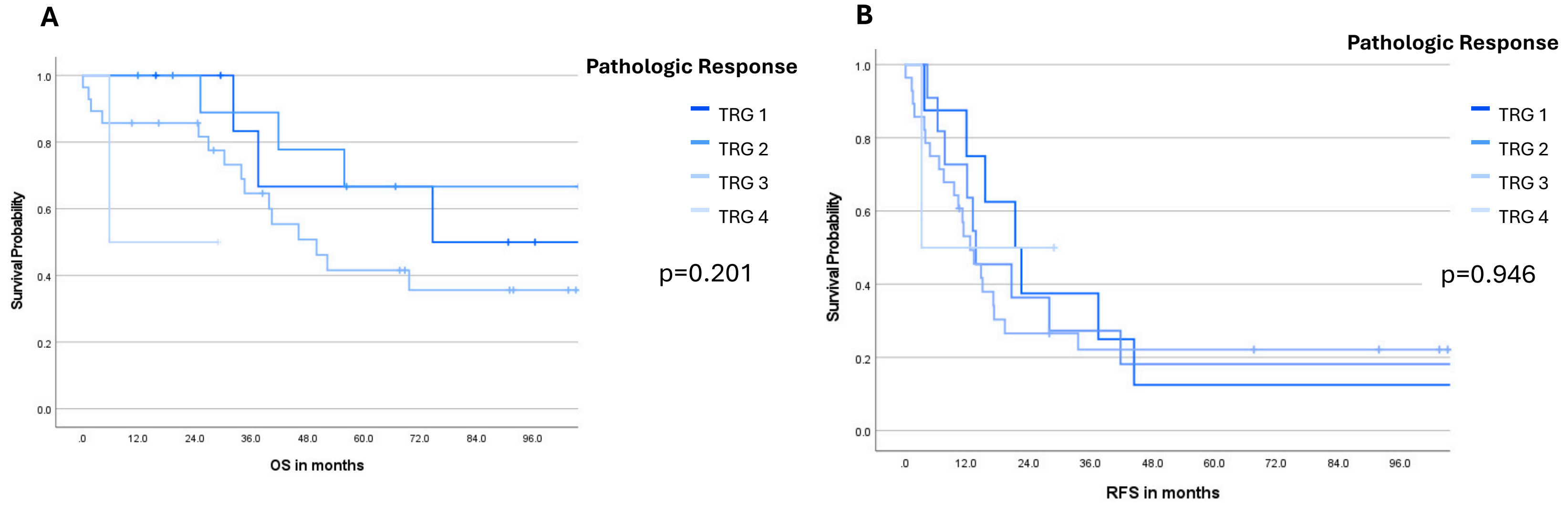

No. at risk

—	8	8	7	5	4	4	4	3	1
—	11	10	9	8	7	5	4	4	4
—	28	23	22	15	11	9	6	6	4
—	2	1	1	0	0	0	0	0	0

No. at risk

—	8	6	3	3	1	1	1	1	1
—	11	8	4	3	2	2	2	2	2
—	28	14	7	5	5	5	4	4	3
—	2	1	1	0	0	0	0	0	0

Supplementary Figure *S*2: Overall survival (OS, panel A) and recurrence-free survival (RFS, panel B) stratified **by pathologic response according to the tumor regression grade (TRG) system by Rubbia-Brandt et al**. No response (TRG 4) vs minor response (TRG 3) vs major response (TRG 2) vs complete response (TRG 1). Numbers at risk are shown below the Kaplan–Meier curves. Statistical significance was defined as a p-value < 0.05.

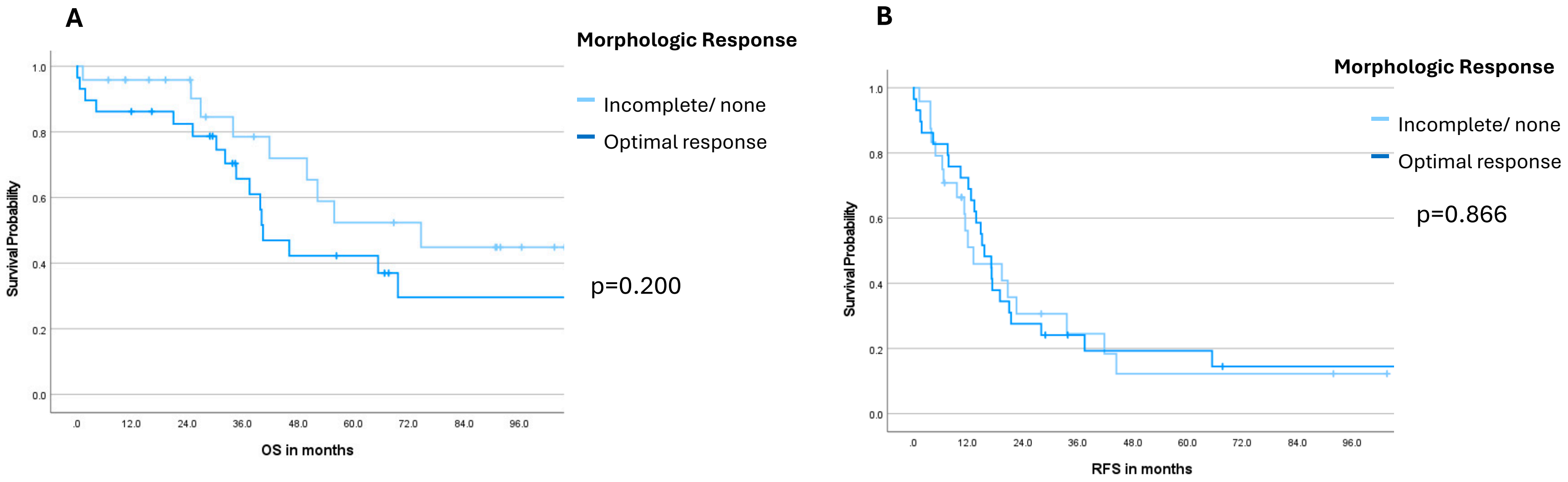

No. at risk (panel A)

Incomplete/ none	24	20	18	13	11	8	7	6	3
Optimal response	29	24	22	14	9	8	4	4	4

No. at risk (panel B)

Incomplete/ none	24	10	6	4	2	2	2	2	1
Optimal response	29	21	8	5	4	4	2	2	2

Supplementary Figure S3. Overall survival (OS, panel A) and recurrence-free survival (RFS, panel B) stratified by **morphologic response** on computed tomography according to the Chun classification. Morphologic response was defined as optimal when the metastasis changed from group 3 or 2 to group 1, incomplete when the group changed from 3 to 2, and absent when the group remained unchanged or increased. Numbers at risk are displayed below the Kaplan–Meier curves. A p-value < 0.05 was considered statistically significant.

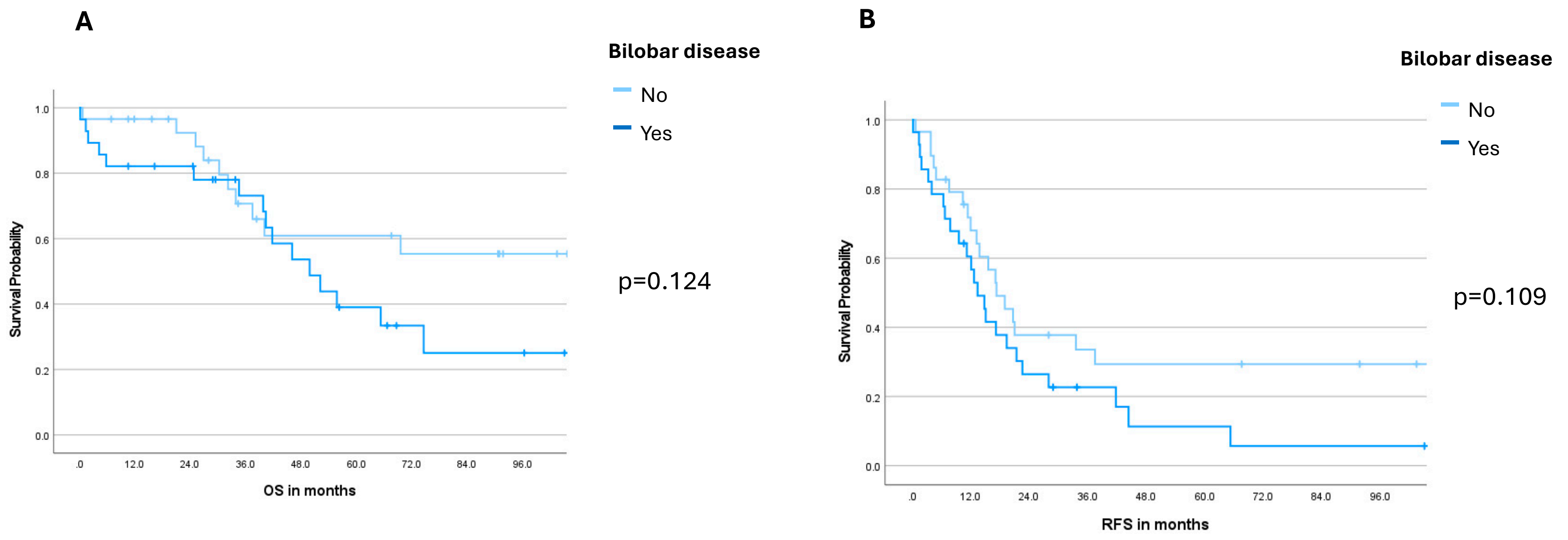

A — No. at risk

No	29	25	22	15	12	12	10	10	7
Yes	28	22	21	15	11	7	4	3	3

B — No. at risk

No	29	18	10	8	7	7	6	6	5
Yes	28	16	7	4	2	2	1	1	1

Supplementary Figure S4. Overall survival (OS, panel A) and recurrence-free survival (RFS, panel B) stratified by **presence of bilobar disease** (yes vs no). Numbers at risk are displayed below the Kaplan–Meier curves. A p-value < 0.05 was considered statistically significant.

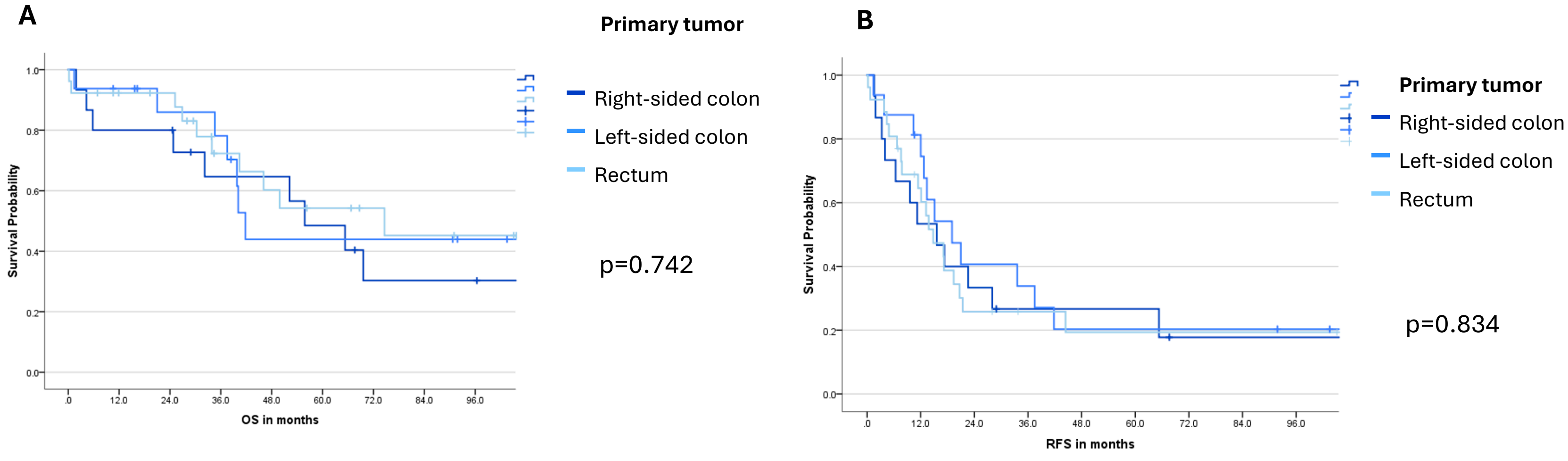

No. at risk (panel A)

Right-sided colon	15	12	12	8	8	6	3	3	3
Left-sided colon	16	14	11	10	5	5	5	5	3
Rectum	26	21	20	12	10	8	6	5	5

No. at risk (panel B)

Right-sided colon	15	8	5	3	3	3	1	1	1
Left-sided colon	16	11	6	5	3	3	3	3	2
Rectum	26	15	6	4	3	3	3	3	3

Supplementary Figure S5. Overall survival (OS, panel A) and recurrence-free survival (RFS, panel B) stratified by the **location of primary tumor** (right-sided colon, left-sided colon, rectum). Numbers at risk are displayed below the Kaplan–Meier curves. A p-value < 0.05 was considered statistically significant.

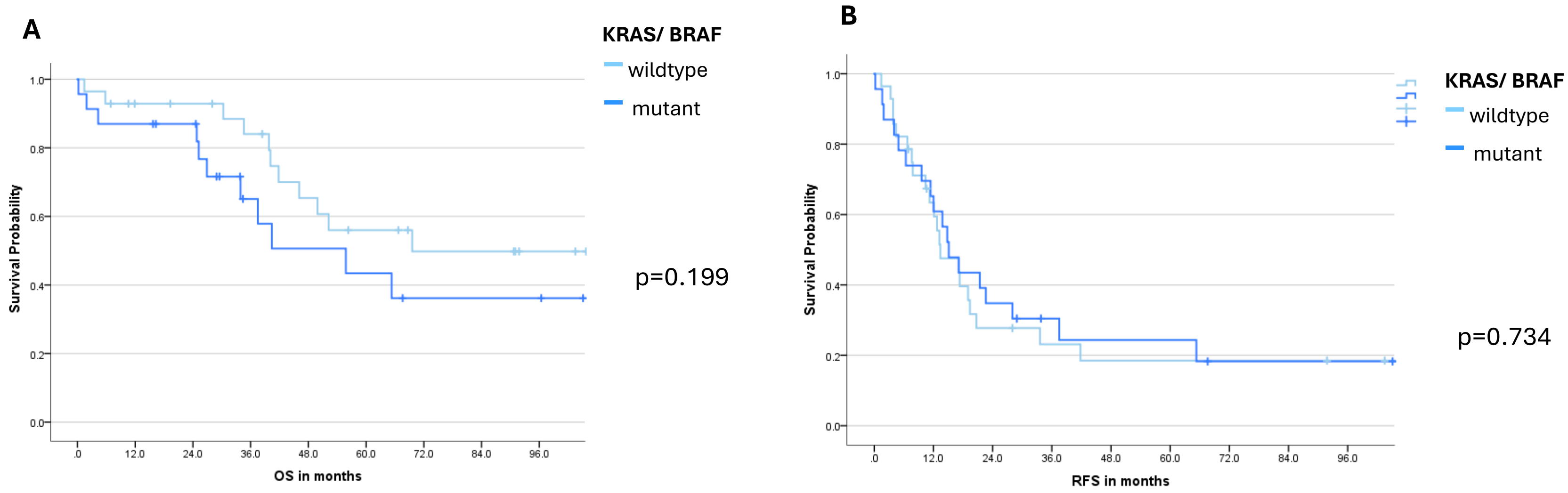

No. at risk (panel A)

wildtype	28	23	22	19	14	11	8	8	5
mutant	23	20	18	9	7	6	4	4	4

No. at risk (panel B)

wildtype	28	16	7	5	4	4	4	4	3
mutant	23	15	8	5	4	4	2	2	2

Supplementary Figure S6. Overall survival (OS, panel A) and recurrence-free survival (RFS, panel B) stratified by the **KRAS or BRAF mutation.** Numbers at risk are displayed below the Kaplan–Meier curves. A p-value < 0.05 was considered statistically significant.
